# Immunotherapy for Alzheimer’s disease: hoops and hurdles

**DOI:** 10.1186/1750-1326-8-36

**Published:** 2013-10-22

**Authors:** Cynthia A Lemere

**Affiliations:** 1 Center for Neurologic Diseases, Brigham and Women’s Hospital, Harvard Medical School, NRB 636F, 77 Avenue Louis Pasteur, Boston 02115, MA, USA

**Keywords:** Immunotherapy, Vaccine, Amyloid-β, Pyroglutamate Aβ, Tau, BACE-1

## Abstract

Alzheimer’s disease (AD) is the most common form of dementia, afflicting more than 30 million people worldwide. Currently, there is no cure or way to prevent this devastating disease. Extracellular plaques, containing various forms of amyloid-β protein (Aβ), and intracellular neurofibrillary tangles (NFTs), composed of hyper-phosphorylated tau protein, are two major pathological hallmarks of the AD brain. Aggregation, deposition, and N-terminal modification of Aβ protein and tau phosphorylation and aggregation are thought to precede the onset of cognitive decline, which is better correlated with tangle formation and neuron loss. Active and passive vaccines against various forms of Aβ have shown promise in pre-clinical animal models. However, translating these results safely and effectively into humans has been challenging. Recent clinical trials showed little or no cognitive efficacy, possibly due to the fact that the aforementioned neurodegenerative processes most likely pre-existed in the patients well before the start of immunotherapy. Efforts are now underway to treat individuals at risk for AD prior to or in the earliest stages of cognitive decline with the hope of preventing or delaying the onset of the disease. In addition, efforts to immunize against tau and other AD-related targets are underway.

## Review

### Alzheimer’s disease: a brief introduction

Alzheimer’s disease afflicts 1 in 9 elderly individuals, and accounts for dementia in more than 5.2 million Americans and more than 30 million people worldwide [1]. The cost for AD care is enormous, with an estimated sum of $200 billion in the USA alone last year. Currently, there is no disease-modifying cure or prevention for AD. The build-up of amyloid-β aggregates, possibly due to slowed clearance, leads to Aβ plaque deposition and vascular amyloid in neocortex and hippocampus years to a decade or more before the onset of clinical symptoms [2]. Subsequently, there is a rise in NFTs and neuron loss that correlates with mild changes in memory. As the pathology progresses, the cognitive impairment becomes more severe, leading to full-blown dementia.

The identification of rare genetic mutations in the amyloid precursor protein (APP), and the presenilins (PS1, PS2), as well as Trisomy 21 (Down syndrome, DS), that invariably lead to AD pathogenesis support the hypothesis that Aβ is an early, key player in the disease [3]. As such, a number of therapeutic strategies targeting Aβ and its downstream consequences are under investigation. These include inhibition or modulation of Aβ-generating proteases by small molecules or immunotherapy, prevention of Aβ aggregation and promotion of Aβ clearance by Aβ immunotherapy, and interference of the toxic response to Aβ by neurons by tau immunotherapy.

### Active versus passive immunization: advantages and disadvantages

Active and passive immunotherapies are currently under investigation for AD. While both strive to slow or prevent cognitive decline, each has its own advantages and disadvantages [4]. Active vaccination, for example, engages the cellular and humoral immune system, including T cells and B cells, to promote the production of anti-antigen antibodies. Typically, an active vaccine is comprised of an antigen (alone or conjugated to a non-self T helper cell epitope) combined with an immune boosting adjuvant to ensure high antibody titers. On one hand, active immunotherapy is attractive because it can induce long-term antibody production in a large population, while being cost-effective and requiring only a few doctor’s visits. However, an active vaccine also induces a T cell response that can increase the risk of a deleterious immune response (i.e., release of pro-inflammatory cytokines), especially, if the T cell recognizes the antigen as a self-protein. And, it takes time to “shut off” an active vaccine immune response. An active vaccine leads to a polyclonal antibody response, which means that it generates antibodies recognizing multiple, sometimes overlapping epitopes on the target protein. This may be helpful for broad coverage or, it may be less useful if the goal is to lower a specific form of a protein but not all forms.

Passive immunotherapy involves the direct injection of monoclonal antibodies (or fragments thereof) without requiring the immune system to generate an antibody response. Several benefits of passive immunotherapy are that it can be stopped immediately if there are any adverse reactions and, that one can target specific epitopes or pathogenic conformations without disturbing the other forms of the protein of interest. On the downside, passive immunization typically requires the production of expensive humanized monoclonal antibodies and monthly injections in a doctor’s office, thereby making it less feasible for long-term treatment of a large population compared to active immunization. In addition, repeated dosing with antibodies over time may lead to the formation of anti-antibodies, which could potentially have a neutralizing effect and/or lead to side effects such as glomerulonephritis and vasculitis.

### Active Aβ immunotherapy

In the mid-1990’s, Beka Solomon’s lab first suggested that anti-Aβ could be used to prevent Aβ fibril formation and disaggregate pre-formed fibrils [5,6]. In 1999, Schenk and colleagues at ELAN Pharmaceuticals demonstrated *in vivo* that active immunization against full-length Aβ with an adjuvant reduced plaque burden in an AD transgenic (Tg) mouse model [7]. Subsequent studies, including our own, demonstrated that active Aβ immunization generates anti- Aβ antibodies that bind human plaques and have B cell epitopes within the first 15 amino acids of the Aβ peptide while the T cell epitopes resided within the mid-region and C-terminus of Aβ [reviewed in 4]. Active and passive Aβ immunotherapy in AD Tg mice were shown to reduce cerebral Aβ and improve cognition, especially if given before the onset of disease pathology (i.e., prophylactically).

The first active vaccine clinical trial for AD, AN1792, was halted early in 2002 due to the development of meningoencephalitis in ~6% (18 of 300) of the enrolled moderate-to-severe AD patients [8]. AN1792 was comprised of full-length Aβ1-42 peptide formulated in a strong adjuvant (QS-21; saporin) and polysorbate 80 to increase solubility of the Aβ1-42 peptide and improve the stability of the vaccine. Most patients received 1–3 doses; approximately 19% made anti- Aβ antibodies (>1:2,000 titer; “responders”) that bound AD plaques and vascular amyloid in human brain sections. While Aβ deposition was reduced focally in specific brain regions in the small number of responders that came to autopsy over the next several years, many were severely demented at the time of death [9], indicating that removal of plaques during late stage AD pathogenesis, after the formation of NFTs and the rampant region-specific loss of neurons, was not beneficial. Interestingly, tau aggregates in neuropil threads and dystrophic neurites, often associated with plaques, were reduced by AN1792 vaccination but no changes were observed in tau accumulation within neuronal cell bodies [10]. The exact cause of the meningoencephalitis in the AN1792 trial is unknown, however, possible causes might include the recognition of the antigen (full-length Aβ peptide) by Aβ-specific T cells, the strong Th1-biased adjuvant, or possibly, the re-formulation of the vaccine with polysorbate 80.

As a result of the AN1792 trial, a large effort was undertaken to test passive immunotherapy using humanized anti- Aβ monoclonal antibodies (mAb), as described in the next section, to avoid any autoimmune-like reactions. In addition, studies were initiated to develop second-generation active vaccines, including many vaccines that target an Aβ B cell epitope, while avoiding Aβ T cell epitopes. These include mimotopes vaccines, neoepitopes vaccines, Aβ-conjugates, and DNA, phage, virus-like particle and adenovirus-associated viral vector vaccines [reviewed in 4].

Currently, several second-generation active Aβ vaccines are being tested in clinical trials (http://www.clinicaltrials.gov). Janssen and Pfizer are conducting Phase II studies to monitor the effects of their Aβ short N-terminus peptide-conjugate vaccine called ACC-001, formulated in the adjuvant QS-21 [11]. Last year, Novartis Pharmaceuticals reported Phase I data for their active Aβ vaccine, CAD106, which consists of multiple copies of Aβ1-6 on QB virus-like particles with or without adjuvant [12]. Phase II CAD106 clinical trials were completed recently and the data analyses are pending. Affiris AG is testing mimotopes, molecular mimics of specific antigen epitopes, against an unmodified Aβ N-terminus (Phase II) and a pyroglutamate-3-modified Aβ N-terminus (Phase Ib) [13]. AC Immune is continuing their combined Phase I/IIa clinical trial to investigate ACI-24, an active Aβ vaccine intended to induce beta-sheet conformation-specific antibodies, similar to a liposomal vaccine against Aβ1-15 that they previously showed in preclinical studies reduced plaques and restored memory [14]. The main goal of these active vaccines is to prevent plaque deposition and/or enhance Aβ clearance.

### Passive Aβ immunotherapy

In 2000, Bard and colleagues first demonstrated that systemic injection of an Aβ monoclonal antibody specific for the Aβ N-terminus, 3D6 mAb, into AD Tg mice resulted in transfer of the antibody into brain, binding of the antibody to plaques, and induction of Fc-receptor-mediated microglial phagocytosis of Aβ deposits [15]. This antibody is the precursor to the humanized N-terminal-specific mAb, Bapineuzumab, which went on to be tested in Phase I, II and III clinical trials. While Bapineuzumab was shown to lower Aβ burden in brain by *in vivo* amyloid PET imaging in a Phase II trial in mild-to-moderate AD patients [16], no significant clinical benefits have been reported in 2 large Phase III clinical trials, according to http://www.clinicaltrials.gov, leading to the termination of other Phase III Bapi trials in 2012. Two possibilities for the lack of clinical efficacy of Bapi include the possibility that not enough antibody made it into the brain and/or that the treatment was too late in the disease process to reverse the neurodegenerative changes that underlie memory loss. Earlier intervention with Aβ immunotherapy may help clarify these points. According to http://www.clinicaltrials.gov, Pfizer and Janssen are currently conducting an Open Label Extension Phase I clinical study in mild-moderate AD patients to test the safety and tolerability of a more recent version of Bapineuzumab, AAB-003, that was re-engineered to reduce the risk of vasogenic edema and microhemorrhage.

Other Aβ mAbs, targeting epitopes in the N-terminus, mid-region, and C-terminus, as well as conformation-specific mAbs, have been tested pre-clinically for their ability to prevent or lower plaque burden and improve cognition [reviewed in 4]. In 2002, Pfeifer et al., reported that repeated administration of an Aβ mAb that recognized Aβ3-6 in old APP23 Tg mice with a high vascular amyloid load resulted in plaque lowering but also significantly increased the number of cerebral microhemorrhages [17]. This was confirmed in other preclinical studies in AD Tg mice [18,19]. Treatment with Bapineuzumab has been associated with transient vasogenic edema and microhemorrhage, especially in AD patients bearing one or two Apolipoprotein E ϵ4 alleles [20].

In 2001, DeMattos and colleagues reported that a mid-region Aβ mAb, that preferentially binds soluble Aβ (m266) lowered brain Aβ burden and increased Aβ levels in plasma, suggesting that the antibodies enhanced clearance from brain to blood [21]. A single injection of m266 mAb was reported to improve cognition within a few days in 2 year-old AD Tg mice [22]. The m266 mAb is the precursor to Lilly’s Solanezumab that is currently in Phase III clinical trials. Solanezumab has not been associated with vasogenic edema or microhemorrhages but does increase plasma Aβ [23]. Recently, Lilly announced that 18 months of treatment with Solanezumab significantly slowed cognitive decline in mild AD patients when they combined cohorts from 2 Phase III studies [24].

Based on the aforementioned clinical trial results, most new Aβ passive trials are focused on prevention and very early treatment of AD. Genentech, the Banner Institute and the National Institutes of Health have partnered to conduct a secondary prevention trial called API (the Alzheimer’s Prevention Initiative) in 300 individuals from a large Colombian family with a mutant gene (PS1 E280A) associated with a dominant form of early onset Alzheimer’s disease. This mutation leads to early and robust cerebral Aβ42 plaque deposition at a relatively young age [25], which is followed within 10–15 years by a progressive decline in cognition and clinical function [26]. Participants 30 years of age and older will be included in this prevention study that will test Genentech’s Crenezumab mAb, licensed from AC Immune. Crenezumab is a humanized Aβ mAb that binds soluble, oligomeric and fibrillar Aβ but unlike the other antibodies tested thus far, Crenezumab was designed on an IgG4 backbone to reduce the risk of microglial-mediated pro-inflammatory effects in brain, including vasogenic edema [27]. The study is expected to begin in 2013.

Other prevention/early treatment trials are expected to begin this year (2013). DIAN, the Dominantly Inherited Alzheimer Network, will conduct a collaborative trial between Lilly, Roche, and the Alzheimer’s Association, in adult children of a parent with a familial Alzheimer’s disease mutant gene that causes dominantly inherited AD. Lilly’s Solanezumb mAb (described above) and Roche’s mAb, Gantenerumab, will be tested. Gantenerumab, which recognizes an epitope in the Aβ N-terminus and then another in its mid-region and preferentially binds fibrillar Aβ [28], is already in Phase III clinical trials in prodromal AD subjects who are amyloid-positive by PET imaging but not yet cognitively impaired. A third prevention trial called A4, Anti-Amyloid Treatment for Asymptomatic Alzheimer’s Disease, will test Solanezumab in 1,000 people 70 years of age and older, without a dominant genetic predisposition to AD, who have positive PET scans for brain amyloid but have not developed clinical AD symptoms.

Additional Aβ passive immunotherapies are also currently under investigation. For example, Eisai Inc. is conducting a large Phase II clinical trial in 800 patients with early AD to study the effects of an Aβ mAb, BAN2401, that recognizes large oligomers (called protofibrils) to try to prevent their toxic effects on neurons. BAN2401 was developed by BioArtic Neuroscience AB and licensed to Eisai in 2007. Biogen Idec is also moving forward with their Aβ humanized IgG1 mAb, BIIB037, that binds strongly to fibrillar Aβ in plaques but less well to vascular amyloid (as reported by Dr. Jeff Sevigny at the AD/PD International Conference, Florence 2013). After a successful single ascending dose Phase I safety study, BIIB037 will soon be tested in prodromal and mild AD patients who are being recruited for a multiple dose Phase 1 study.

Pre-clinical studies have reported beneficial effects of passive immunotherapy against other Aβ-related targets as well. For example, pyroglutamate-3 Aβ is a highly pathogenic Aβ species found in plaques and vascular amyloid but not in CSF or plasma, that may act as a seed for Aβ aggregation [29,30]. We reported that a pyroglutamate-3 β mAb, 07/1, provided by our collaborators at Probiodrug AG, reduced plaque burden in young and old AD Tg mice, in the absence of increased vascular amyloid or microhemorrhage [31]. Others have reported similar findings [32,33]. Recently, we found that the anti-pyroglu Aβ 07/1 mAb partially spared cognitive deficits in an AD Tg mouse model (as reported by Jeff Frost at the AD/PD Conference in Florence, Italy in March 2013). It is likely that a pyroglutamate-3 Aβ mAb will not get saturated by binding to Aβ in blood, thereby potentially enhancing the transfer of the antibody into brain to prevent Aβ deposition into plaques and blood vessels and to enhance Aβ clearance.

### Alternative AD immunotherapies: IVIg and tau immunotherapy

Intravenous immunoglobulin (IVIg), pooled human antibodies, showed promise in early (pilot and Phase II) clinical trials reviewed in [34]. However, recent trials, including Octapharma USA’s Phase II 24-week Octagam 10% IVIg trial in 58 AD patients [35] and Baxter Healthcare Corporation’s large 18-month Phase III Gammagard 10% IVIg trial in 390 mild-moderate AD [36] showed no significant slowing of AD progression. As a result, Baxter has terminated its IVIg program for Alzheimer’s disease. Ongoing IVIg clinical trials include a small Phase II study of Octagam by Sutter Health Neuroscience Institute in 50 MCI subjects and a Phase III study by Grifols Biologicals, Inc. in which 350 mild-moderate AD subjects are being treated with a combination of albumin and IVIg. Both trials are due to be completed in 2014, according to http://www.clinicaltrials.gov.

Lastly, the interest in tau immunotherapy for AD and tau-relevant neurodegenerative diseases has grown immensely over the past few years, perhaps in part, due to the failure of Aβ IT to *reverse* cognitive deficits in moderate-severe AD patients. At least 8 preclinical reports have been published to date regarding the beneficial effects of active and passive immunotherapy targeting tau aggregates and/or tau phospho-epitopes in tau Tg mouse models reviewed in [37]. Axon Neuroscience SE (Graz, Austria) recently began recruiting mild-moderate AD patients for a Phase I safety study of their new AADvac1 tau-peptide-KLH-conjugate active vaccine, formulated in alum (http://www.clinicaltrials.gov). It is likely that other active as well as passive tau vaccines will be forthcoming.

### Challenges for future AD immunotherapies

While the field of AD immunotherapy has grown tremendously over the past 10–13 years, certain issues still remain and may need to be overcome in order to see long-term, clinical safety and efficacy. First, more antibodies, whether generated by active immunization or administered passively, may need to get into the brain to be effective. Typically, only a small percentage of antibodies cross the blood brain barrier (~0.1%), thus it may be helpful to find ways to improve antibody penetration into brain. Some possibilities include: the use of chaperone proteins or bi-specific antibodies to ferry the therapeutic antibodies into brain, transient opening of the BBB by chemical or radiological means, and direct infusion of antibodies into the CNS using a time-released pump. Second, removing amyloid after neurons are lost has not been effective thus far, suggesting that treatment should be started earlier and perhaps, should be tested for longer periods of time. Such studies are now underway. Third, a better understanding of the clearance of Aβ/anti-Aβ immune complexes is needed to avoid clogging of the clearance pathway during long-term treatment. Active vaccination requires particular attention to immune effects of immunotherapy, including immunosenescence in the elderly, the potential for autoimmune effects when vaccinating against self-proteins, and the use of strong, pro-inflammatory adjuvants. Vaccine platforms that have been shown to safely generate reasonable titers in the elderly and immuno-compromised humans may be useful for an AD vaccine. For example, we recently utilized Mercia Pharmaceutical’s MER vaccine platform, which was shown previously to safely generate titers against self-proteins in two cancer vaccines, to test an Aβ1-15:diptheria toxoid conjugate vaccine called MER5101, which was formulated in an adjuvant, MAS-1, in an AD transgenic mouse model. The vaccine generated high titers and lowered plaques, induced an anti-inflammatory immune response, and improved cognition [38]. And lastly, improving the sensitivity of biomarkers, including imaging of pre-amyloid diffuse plaques, and cognitive/functional tests to detect the earliest changes in AD will allow for better patient selection for clinical trials and more sensitive outcome measures.

## Conclusions

It is now well accepted that Alzheimer’s disease pathogenesis begins years, if not decades, before the onset of clinical symptoms. Aβ aggregation and accumulation, as well as N-terminal truncation and modification, are very early, region-specific events in AD. Genetics, CSF biomarkers and imaging of brain structure and amyloid deposition help predict individuals at risk for developing AD. Thus far, amyloid-lowering treatments (e.g., active and passive Aβ immunotherapy) have shown little or no cognitive benefit in moderate to severe AD patients, in whom the disease process had been underway for years. This suggests that removing amyloid cannot reverse cognitive deficits once significant neuronal damage has occurred. Instead, the aim now is to start Aβ immunotherapy at the onset of AD pathological changes, prior to or in the very early stages of clinical symptoms, in the hope of preventing downstream events, such as neuroinflammation and tau pathology, that lead to neuron loss and cognitive impairment. Tau immunotherapy, especially vaccines targeting pathogenic forms of tau protein, may be effective in slowing cognitive decline once AD pathogenesis is underway and/or has manifested in cognitive changes. Taken together, it remains possible that with earlier detection and treatment, it may be possible to prevent or delay Alzheimer’s disease in the future. Considering the growing medical, economic, and societal burden of AD, the need for an effective treatment is stronger than ever.

## Abbreviations

Aβ: Amyloid-β protein; AD: Alzheimer’s disease; APP: Amyloid-β precursor Protein; CSF: Cerebral spinal fluid; DS: Down syndrome; IT: Immunotherapy; MRI: Magnetic resonance imaging; NFT: Neurofibrillary tangle; PS1: Presenilin 1; PS1: Presenilin 2; Tg: Transgenic.

## Competing interests

The author is an unpaid Scientific Advisor to Probiodrug AG, Halle, Germany, which provides Aβ monoclonal antibodies for her preclinical research.

## Author’s contributions

CAL conceived of and drafted the manuscript, which reflects her view of the field at this time.

## Author’s information

CAL is an Associate Professor of Neurology at the Brigham and Women’s Hospital and Harvard Medical School in Boston, MA. She has performed preclinical studies of Aβ immunotherapy in AD-like transgenic mouse models and aged non-human primates for more than 14 years.
